# Prognostic significance of the TyG index combined with the NLR for heart failure: a retrospective study

**DOI:** 10.3389/fcvm.2026.1712467

**Published:** 2026-04-21

**Authors:** Sirui Yang, Hongxing Zhang, Yanqing Liu, Lusha Gao, Yun Song, Ping Xia, Tao Shi, Fazhi Yang, Lixing Chen

**Affiliations:** Department of Cardiology, Kunming Medical University First Affiliated Hospital, Kunming, Yunnan, China

**Keywords:** heart failure, mortality, neutrophil-to-lymphocyte ratio, prognostic, triglyceride glucose index

## Abstract

**Background:**

The triglyceride–glucose (TyG) index and the neutrophil–lymphocyte ratio (NLR) are independent prognostic factors in patients with heart failure, but no studies have explored the predictive value of the TyG index combined with the NLR (TyG-NLR) for all-cause mortality in patients with heart failure.

**Methods:**

A total of 1,063 patients with HF admitted to the First Affiliated Hospital of Kunming Medical University from January 2017 to October 2021 were enrolled in the study. Based on the median of TyG-NLR, patients were divided into a low TyG-NLR group (TyG-NLR < 5.93) and a high TyG-NLR group (TyG-NLR ≥ 5.93) and 4 subgroups according to the median TyG and NLR (Group 1: TyG <1.79 + NLR < 3.31; Group 2: TyG < 1.79 + NLR ≥ 3.31; Group 3: TyG ≥ 1.79 + NLR < 3.31; Group 4: TyG ≥ 1.79 + NLR ≥ 3.31). We used Kaplan–Meier curves, Cox survival analyses, ROC curves, NRI, IDI, and DCA to explore the predictive value of the TyG-NLR for all-cause mortality in HF patients.

**Results:**

According to the Kaplan–Meier analysis, those with higher TyG indices and NLRs (TyG ≥ 1.79 and NLR ≥ 3.31) had significantly higher mortality rates than the other patients. By univariate and multivariate Cox proportional hazards analyses, we identified the TyG-NLR as an independent predictor of all-cause mortality in patients with CHF. In the subgroup analysis, the risk of death and all-cause mortality rates were 2.033 times higher in Group 2 (*p* < 0.001), 1.486 times higher in Group 3 (*p* = 0.018), and 2.984 times higher in Group 4 (*p* < 0.001), with Group 1 being the reference group. In addition, the receiver operating characteristic (ROC) curves, net reclassification improvement (NRI), integrated discrimination improvement (IDI), and decision curve analysis (DCA) analysis revealed that the model with the TyG-NLR is superior to the traditional model in predicting all-cause mortality in patients with HF.

**Conclusions:**

Our findings demonstrate that an increased TyG-NLR is an independent predictor of increased mortality in patients with HF.

## 1 Introduction

Epidemiological studies have shown that heart failure (HF) is a growing global public health burden, with 64.3 million people estimated to suffer from HF worldwide (1). Despite the enormous progress in therapy and tool development in recent decades to predict incidence and adverse outcomes, the incidence of HF has continued to rise dramatically over time (2). The prevention of disease and death due to heart failure is a global health priority. Therefore, identifying more specific predictors of future HF events and identifying high-risk groups for heart failure is very important for timely intervention and effective prevention strategies (3).

Insulin resistance (IR), a marker of metabolic disorders and systemic inflammation, is an independent and significant risk factor for HF and cardiovascular (CV) death (4–6). Several methods to assess IR have been developed, and among these methods, the homeostasis model assessment of insulin resistance (HOMA-IR) and the triglyceride-glucose (TyG) index are considered valuable and reliable markers of IR (7, 8). The TyG index, a metric derived from fasting plasma glucose (FPG) and triglyceride (TG) levels, was first proposed by Unger et al. in 2013 as an alternative indicator of IR (9). It has been proposed as a reliable surrogate marker of insulin IR (10). An increasing number of studies have reported a positive correlation between the TyG index and cardiovascular diseases such as stroke, myocardial infarction, HF, hypertension and arterial stiffness (11–15). Previous studies have shown that the TyG index may play an essential role in the impairment of left ventricular structure and function (16, 17) and is associated with the development of HF and poor prognosis (18–21).

Inflammation plays a pivotal role in HF development and disease progression, promoting fibrosis and remodeling through different mechanisms. Neutrophils and lymphocytes are the two main arms of inflammation, and the recent combination of these two blood markers makes for a great indicator of inflammation, the neutrophil-to-lymphocyte ratio (NLR), which has been reported to be a useful prognostic tool in cardiovascular disease (22, 23). Due to the imbalance between inflammatory and anti-inflammatory pathways in HF, decreased neutrophil apoptosis leads to elevated absolute counts and an increased incidence of HF (24, 25). On the other hand, the decompensated state of HF leading to decreased lymphocyte counts and lymphopenia has been shown to be an independent predictor of death in HF (26). The NLR combines two distinct immune pathways: a rapid response involving neutrophils and a long-term adaptive response of the immune system associated with lymphocytes (22), and blood counts are readily available in patients with HF, making this valuable for risk stratification of the HF patient population.

Recently, an increasing number of studies have integrated multiple indicators into novel composite scores to comprehensively evaluate the risk of cardiac metabolism and predict adverse cardiovascular outcomes. There are no studies on the predictive value of the TyG score combined with the NLR for all-cause mortality in patients with HF. The aim of this study was to investigate the correlation between the TyG score combined with the NLR and the long-term prognosis of HF patients, clarify the potential predictive value of this composite indicator for all-cause mortality in this population, and provide new insights for the clinical prognostic risk stratification of HF patients.

## 2 Materials and methods

### 2.1 Study population

We retrospectively analyzed 1,221 decompensated heart failure patients hospitalized at the First Affiliated Hospital of Kunming Medical University from January 2017 to October 2021 according to the following inclusion criteria: admitted with HF (NYHA class III or IV) and BNP (brain natriuretic peptide) levels ≥500 pg/mL. The exclusion criteria were as follows: (1) missing data; (2) had other serious medical conditions: active malignancy, systemic inflammatory or autoimmune diseases (e.g., rheumatoid arthritis, lupus), severe hepatic insufficiency (Child classification III), severe renal insufficiency (creatinine clearance ≤20%); and (3) were lost to follow-up. We ultimately included 1,063 patients with HF in our study.

### 2.2 Data collection and definitions

On admission, we collected demographic and clinical information, blood samples, electrocardiograms, and cardiac ultrasound data from CHF patients. Baseline clinical data included patient age, sex, heart rate (HR), body mass index (BMI), blood pressure (BP), New York Heart Association cardiac function classification (NYHA class), medical history, white blood cell (WBC) count, neutrophil (NBC) count, lymphocyte (LBC) count, neutrophil–lymphocyte ratio (NLR), red blood cell (RBC) count, hemoglobin (Hb), albumin (Alb), platelet (PLT), potassium, chlorine, brain natriuretic peptide (BNP), alanine aminotransferase (ALT), aspartate aminotransferase (AST), uric acid (UA), FPG, total cholesterol (TC), TG, low density lipoprotein cholesterol (LDL-C), High density lipoprotein cholesterol (HDL-C), estimated glomerular filtration rate (eGFR), left ventricular ejection fraction (LVEF), QRS width, left atrial diameter (LAd), left ventricular end-diastolic diameter (LVDd), right atrial diameter (RAd) and right ventricular diameter (RVd). Blood samples were taken from patients after approximately 8–12 h of fasting and submitted to the laboratory of the First Affiliated Hospital of Kunming Medical University.

The researchers collected survival data from the patients by interviewing them or their families over the phone. If no response was received, follow-up was terminated at the time of the patient's last available medical record. The endpoint of this study was all-cause mortality in patients with HF.

The neutrophil-to-lymphocyte ratio (NLR) and triglyceride glucose index (TyG) were measured after admission using the following formula: Triglyceride glucose index (TyG): ln [fasting triglycerides (mmoml/L) × fasting glucose (mmoml/L)/2], neutrophil-to-lymphocyte ratio (NLR): neutrophils (×10^9^/L)/Lymphocytes (×10^9^/L). TyG was combined with the NLR (TyG-NLR): Product of TyG and the NLR.

### 2.3 Statistical analysis

We calculated the medians of the TyG, NLR, and TyG-NLR to be 1.79, 3.31 and 5.93, respectively. Patients with HF were divided into a low TyG-NLR group (TyG-NLR < 5.93) and a high TyG-NLR group (TyG-NLR ≥ 5.93), according to the median TyG-NLR value. And we divided the patients into 4 groups: Group 1 (TyG <1.79 + NLR < 3.31), Group 2 (TyG < 1.79 + NLR ≥ 3.31), Group 3 (TyG ≥ 1.79 + NLR < 3.31) and Group 4 (TyG ≥ 1.79 + NLR ≥ 3.31) based on the median TyG and NLR. To describe the baseline characteristics of patients, continuous variables are expressed as the mean ± standard deviation if normally distributed and as the median with interquartile range if skewed, and categorical variables are expressed as frequencies and percentages.

Variables for the multivariable Cox models were selected based on clinical relevance and univariate association with the outcome (*p* < 0.10). Collinearity among predictors was assessed using the variance inflation factor (VIF); a VIF < 5 was considered acceptable, indicating no severe multicollinearity. In our cohort, the proportion of missing data for key variables (TyG index and NLR) was minimal, and we conducted a full-case analysis.

The data were analyzed statistically via SPSS ver. 25.0 and R 4.4.1. A two-sided *p* value <0.05 was considered statistically significant. This study is exploratory and aims to generate hypotheses for future validation.

## 3 Results

### 3.1 Baseline patient characteristics

In this study, we ultimately enrolled 1,063 patients [aged 66.89 ± 12.51 years, 652 (61.3%) males] with HF. During a median (p25%–p75%) follow-up of 759 (340–1,132) days, 494 (46.47%) patients died. Based on the median TyG-NLR, we divided the patients into two groups: the low TyG-NLR group, with a TyG-NLR < 5.93, and the high TyG-NLR group, with a TyG-NLR ≥ 5.93.

Table 1 shows the baseline characteristics of patients stratified by the TyG-NLR. Compared with those in the low TyG-NLR group, the patients in the high TyG-NLR group tended to be older and male and had higher neutrophil and WBC counts; higher Fib, C-reactive protein, blood potassium, BNP, AST, creatinine, FBG, TG, TC and LDL-C levels; lower lymphocyte counts; lower Hb, blood sodium, chloride, GFR and HDL-C levels; a greater proportion of Killip class > 3; and a greater occurrence of diabetes and coronary heart disease.

**Table 1:** Baseline characteristics according to the TyG-NLR subgroup.

	Total (*n* = 1,063)	Low TyG-NLR group	High TyG-NLR group	*p* value
		(*n* = 531)	(*n* = 532)	
Clinical demographics				
Age (years)	66.89 ± 12.51	61.35 ± 12.40	72.43 ± 9.89	<0.001
Male, *n* (%)	652 (61.3)	315 (59.3)	337 (63.3)	0.178
BMI (kg/m^2^)	23.03 ± 3.83	23.07 ± 3.92	22.98 ± 3.73	0.351
SBP (mmHg)	122.37 ± 23.33	122.54 ± 22.28	122.2 ± 24.36	0.141
DBP (mmHg)	76.38 ± 15.25	77.24 ± 15.63	75.52 ± 14.81	0.061
NYHA functional class III, *n* (%)	667 (62.7)	363 (68.4)	304 (57.1)	<0.001
Medical history				
Smoking status, *n* (%)	362 (34.1)	180 (33.9)	182 (34.2)	0.914
Drinking status, *n* (%)	180 (16.9)	99 (18.6)	81 (15.2)	0.137
Diabetes, *n* (%)	303 (28.5)	104 (19.6)	199 (37.4)	<0.001
Hypertension, *n* (%)	590 (55.5)	284 (53.5)	306 (57.5)	0.186
Coronary heart disease, *n* (%)	538 (50.6)	221 (41.6)	317 (59.6)	<0.001
Atrial fibrillation, *n* (%)	356 (33.5)	191 (36.0)	165 (31.0)	0.087
LVEF				0.037
HFrEF, *n* (%)	438 (41.2)	239 (45.0)	199 (37.4)	
HFmrEF, *n* (%)	214 (20.1)	97 (18.3)	117 (22.0)	
HFpEF, *n* (%)	411 (38.7)	195 (36.7)	216 (40.6)	
Laboratory data				
WBC (10^9^/L)	7.90 (5.56, 8.99)	6.55 (5.18, 7.52)	9.24 (6.29, 10.75)	<0.001
Neutrophils (10^9^/L)	5.48 (3.56, 6.36)	3.96 (3.07, 4.56)	7.01 (4.54, 8.39)	<0.001
Lymphocytes (10^9^/L)	1.49 (1.04, 1.83)	1.78 (1.32, 2.10)	1.19 (0.83, 1.49)	<0.001
RBCs (10^12^/L)	4.55 ± 0.77	4.64 ± 0.75	4.45 ± 0.78	0.228
Hb (g/L)	138.53 ± 23.99	141.57 ± 22.73	135.5 ± 24.85	0.02
PLTs (10^9^/L)	201.70 (150.00, 244.00)	197.35 (151.00, 238.00)	206.04 (149.25, 250.00)	0.07
Fib (g/L)	3.58 (2.73, 4.17)	3.33 (2.66, 3.84)	3.83 (2.85, 4.60)	<0.001
Alb (g/dL)	36.67 ± 4.56	37.44 ± 4.30	35.92 ± 4.69	0.28
CRP (mg/L)	21.52 (2.8, 21.5)	12.82 (2.22, 13,2)	30.20 (4.29, 32.88)	<0.001
LogBNP	3.16 ± 0.28	3.13 ± 0.26	3.20 ± 0.29	0.009
Potassium (mmol/L)	3.94 ± 0.60	3.93 ± 0.57	3.94 ± 0.63	0.044
Sodium (mmol/L)	141.03 ± 4.44	141.77 ± 4.10	140.23 ± 4.65	0.002
Chlorine (mmol/L)	102.93 ± 4.68	103.63 ± 4.34	12.24 ± 4.91	0.002
ALT (IU/L)	44.03 (16.7, 42,5)	37.84 (16.70, 41.00)	50.21 (16.7, 44.78)	0.137
AST (IU/L)	45.64 (20.00, 43.00)	35.89 (19.90, 38.20)	55.36 (21.00, 52,78)	<0.001
Cre, (*μ*mol/L)	121.83 (83.20, 134.10)	109.42 (80.20, 120.20)	134.22 (86.43, 147.40)	<0.001
UA (umol/L)	494.73 (374.40, 588.56)	490.65 (372.53, 546.55)	498.89 (377.88, 602.83)	0.5858
GFR (mL/min)	45.21 (32.18, 56.51)	49.78 (36.46, 60.79)	40.65 (27.72, 51.65)	<0.001
FBG (mmol/L)	6.10 (4.19, 6.50)	5.26 (4.05, 5.60)	6.93 (4.50, 7.70)	<0.001
TC (mmol/L)	3.65 ± 1.01	3.62 ± 0.96	3.68 ± 1.06	0.043
TG (mmol/L)	3.65 (2.95, 4.25)	1.18 (0.83, 1.39)	1.39 (0.94, 1.64)	<0.001
HDL-C (mmol/L)	0.99 (0.79, 1.16)	1.02 (0.82, 1.19)	0.96 (0.76, 1.12)	<0.001
LDL-C (mmol/L)	2.30 (1.67, 2,82)	2.23 (1.65, 2.71)	2.38 (169, 2.99)	0.008
Treatment				
CRT/ICD, *n* (%)	104 (9.8)	51 (9.6)	53 (10.0)	0.844
SGLT2i, *n* (%)	238 (22.4)	108 (20.3)	130 (24.4)	0.109
Beta blockers, *n* (%)	746 (70.2)	378 (71.2)	368 (34.6)	0.473
ACEI/ARB/ARNI, *n* (%)	603 (56.7)	304 (57.3)	299 (56.2)	0.73
Diuretics, *n* (%)	860 (80.9)	386 (72.7)	474 (89.1)	0.543
Aldosterone antagonist, *n* (%)	825 (77.6)	385 (72.5)	440 (82.7)	0.987

BMI, body mass index; SBP, systolic blood pressure; DBP, diastolic blood pressure; NYHA, New York Heart Association cardiac function classification; LVEF, left ventricular ejection fraction; BNP, brain natriuretic peptide; WBCs, white blood cells; RBC, red blood cells; Hb, hemoglobin; PLT, platelet; Fib, fibrinogen; Alb, albumin; CRP, C-reactive protein; BNP, brain natriuretic peptide; ALT, alanine aminotransferase; AST, aspartate aminotransferase; Cre, creatinine; UA, uric acid; GFR, glomerular filtration rate; FBG, fasting blood glucose; TC, total cholesterol; TG, triglyceride; HDL-C, high-density lipoprotein cholesterol; LDL-C, low-density lipoprotein cholesterol; CRT, cardiac resynchronization therapy; ICD, implantable cardioverter-defibrillator; SGLT2i, Sodium-glucose cotransporter-2 inhibitors; ACEI, angiotensin converting enzyme inhibitor; ARB, angiotensin II receptor blocker; ARNI, angiotensin receptor-neprilysin inhibitor.

There were no significant differences in sex, BMI, BP, smoking status, drinking history, or history of hypertension (all *P* < 0.05).

### 3.2 Associations of the TyG index, NLR, and TyG-NLR with all-cause mortality in HF patients

**Figure 1:**
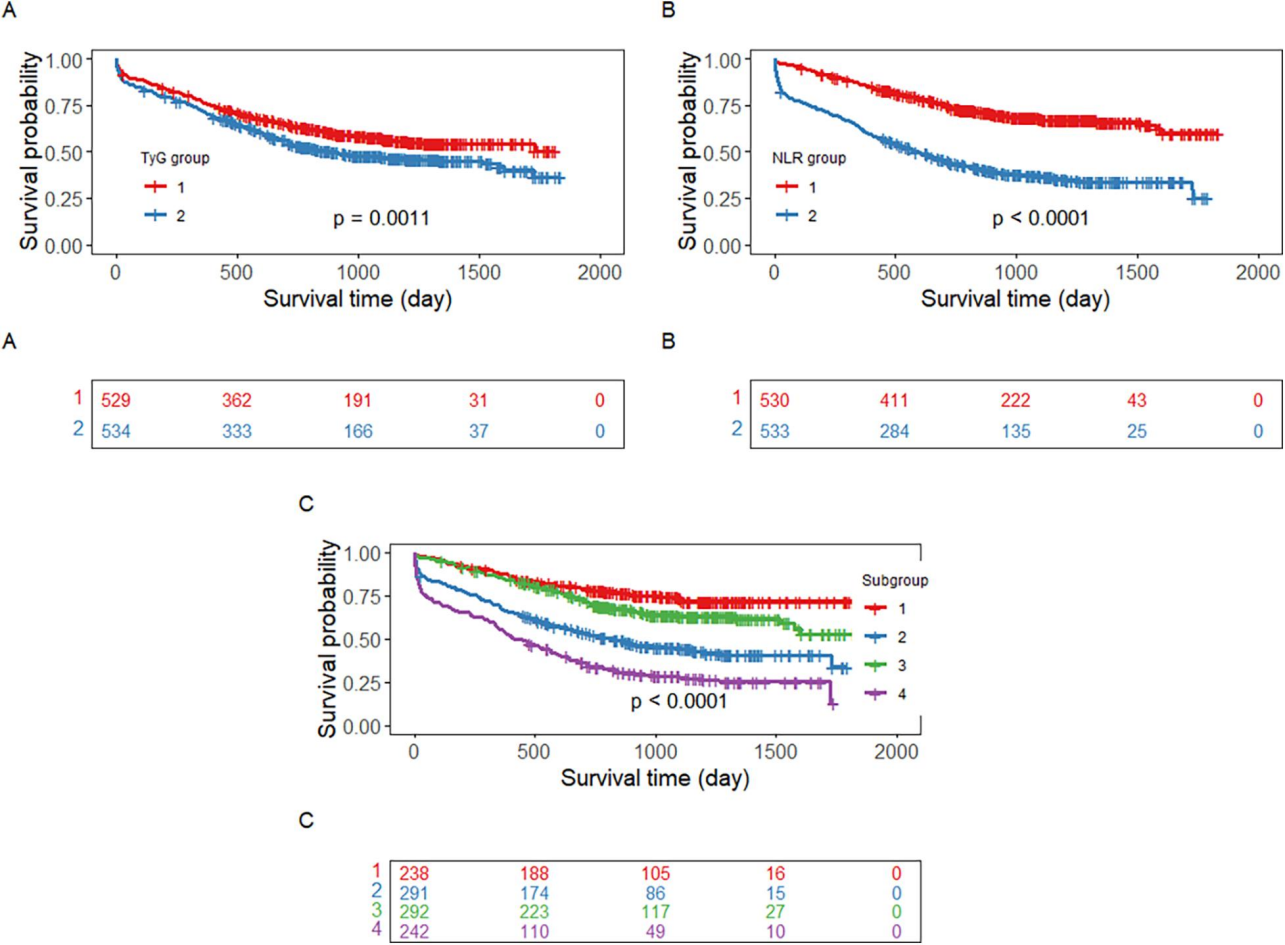
Kaplan–meier survival estimates for all-cause mortality based on **(A)** TyG levels, **(B)** NLR levels, (C. Subgroups of TyG-NLR. **(A)** Group 1: TyG < 1.79; Group 2: TyG ≥ 1.79. **(B)** Group 1: NLR < 3.31; Group 2: NLR ≥ 3.31. **(C)** Group 1: TyG <1.79 + NLR < 3.31; Group 2: TyG < 1.79 + NLR ≥ 3.31; Group 3: TyG ≥ 1.79 + NLR < 3.31; Group 4: TyG ≥ 1.79 + NLR ≥ 3.31.

We analyzed Kaplan–Meier survival curves stratified by the median TyG index (1.79) and NLR (3.31) separately. The cumulative probability of all-cause mortality, as shown by the Kaplan–Meier survival curves, was significantly greater in the high group than in the low group in both the TyG index and NLR groups (Figures 1A,B). As expected, when we combined the TyG index and NLR (TyG-NLR), Kaplan–Meier survival curves revealed that among the four groups, those with higher TyG indices and NLRs (TyG ≥ 1.79 and NLR ≥ 3.31) had significantly higher mortality rates than the other groups did (*p* < 0.0001) (Figure 1C).

### 3.3 TyG-NLR as a predictor of all-cause mortality

The univariable proportional hazards models (Table 2) revealed that the TyG-NLR was independently correlated with all-cause mortality in HF patients. An increase in the TyG-NLR was associated with a 2.531% greater risk of all-cause mortality (95% CI: 2.101–3.549, *P* < 0.001), with the low TyG-NLR group used as a reference. After adjustment for confounding factors, multivariate Cox proportional hazards analysis suggested that the TyG-NLR was still independently correlated with all-cause mortality in HF patients (HR: 1.349, 95% CI: 1.123–1.733; *P* < 0.001).

**Table 2:** Univariable and multivariable analyses with Cox proportional hazards models for all-cause mortality in HF patients.

	Univariable		Multivariable	
	HR (95% CI)	*p*	HR (95% CI)	*p*
TyG-NLR				
Low-TyG-NLR	Ref.		Ref.	
High-TyG-NLR	2.531 (2.101, 3.049)	<0.001	1.394 (1.123, 1.733)	*0*.*003*
Age	1.034 (10.25, 1.042)	<0.001	1.019 (1.008, 1.029)	<0.001
Male (reference: Female)	1.045 (0.871, 1.254)	<0.001		
BMI	0.954 (0.931, 0.977)	<0.001		
Coronary heart disease	1.188 (0.996, 1.418)	0.056		
Hypertension	1.066 (0.892, 1.273)	0.481		
Diabetes mellitus	1.474 (1.224, 1.774)	<0.001		
NYHA functional class III–IV	2.405 (2.016, 2.871)	<0.001	1.840 (1.524, 2.222)	<0.001
LVEF	0.996 (0.991, 1.002)	0.199		
LgBNP	5.456 (3.905, 7.623)	<0.001	2.745 (1.856, 4.059)	<0.001
WBCs	1.092 (1.071, 1.114)	<0.001		
RBCs	0.713 (0.632, 0.804)	<0.001		
HB	0.989 (0.985, 0.933)	<0.001		
PLTs	0.998 (0.997, 0.999)	0.001	0.998 (0.997, 0.999)	0.003
Fib	1.095 (1.023, 1.172)	0.009		
Potassium	1.146 (0.987, 1.332)	0.074		
Chloride	0.922 (0.905, 0.940)	<0.001	0.949 (0.927, 0.972)	<0.001
Sodium	0.929 (0.910, 0.948)	<0.001		
Alb	0.921 (0.902, 0.940)	<0.001	0.945 (0.922, 0.967)	<0.001
ALT	1.003 (1.002, 1.004)	<0.001		
AST	1.005 (1.004, 1.006)	<0.001	1.004 (1.001, 1.006)	0.002
Cre	1.003 (1.002, 1.004)	<0.001		
UA	1.001 (1.001, 1.002)	<0.001		
GFR	0.973 (0.968, 0.978)	<0.001		
CRP	1.011 (1.009, 1.013)	<0.001	1.007 (1.005, 1.010)	<0.001

BMI, body mass index; LVEF, left ventricular ejection fraction; BNP, brain natriuretic peptide; WBCs, white blood cells; RBC, red blood cells; Hb, hemoglobin; PLT, platelet; Fib, fibrinogen; Alb, albumin; ALT, alanine aminotransferase; AST, aspartate aminotransferase; Cre, creatinine; UA, uric acid; GFR, glomerular filtration rate; CRP, C-reactive protein; HR: hazard ratio, CI, confidence interval.

### 3.4 Predictive ability of the TyG-NLR in HF patients

**Figure 2:**
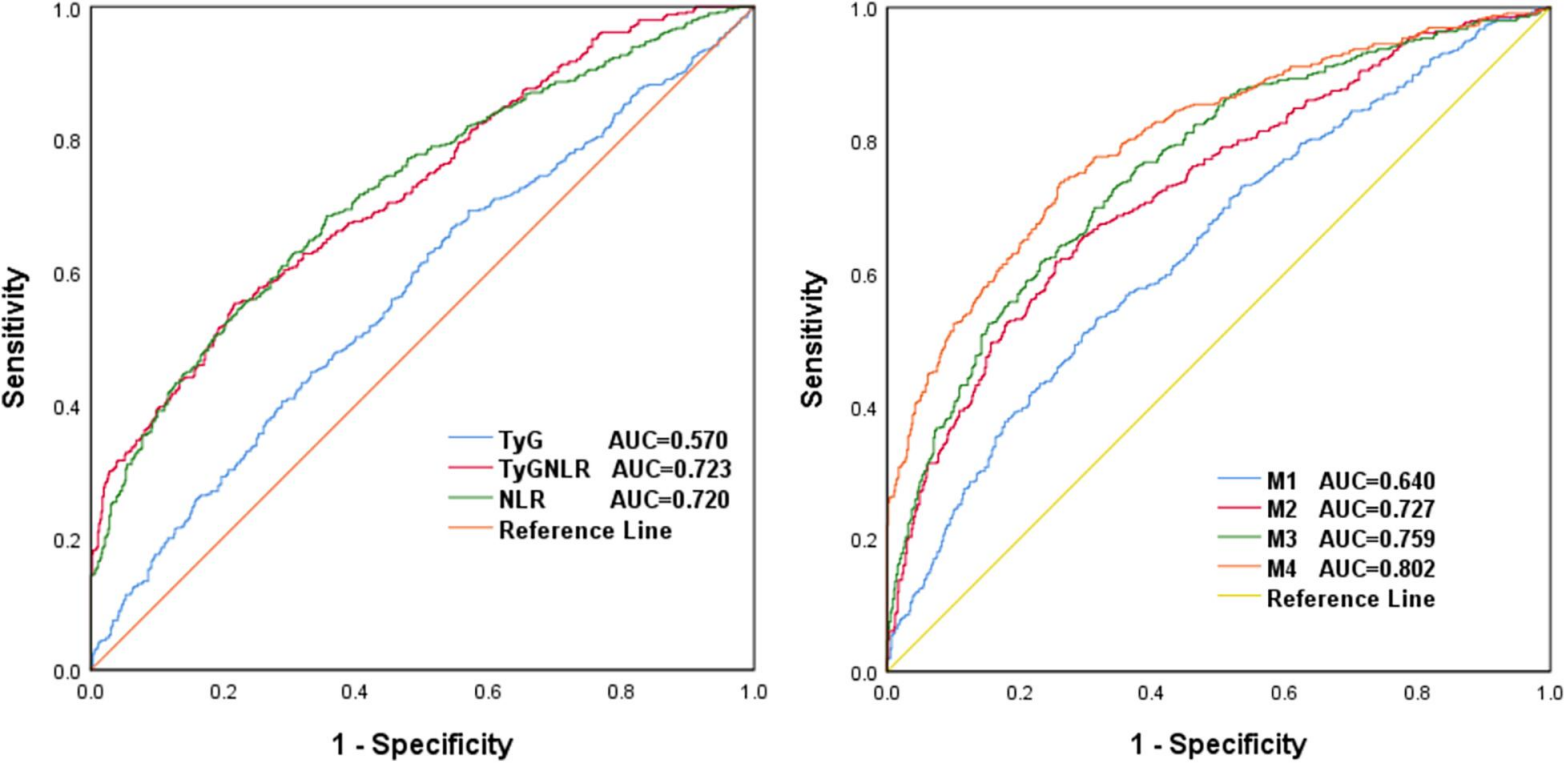
ROC curves of **(A)** TyG, NLR and TyG-NLR and **(B)** the four models for predicting mortality in HF patients. **(B)** Model 1: Adjusted for age, sex and BMI; Model 2: Adjusted for M1+ serum chloride, sodium, albumin and fibrinogen; Model 3: Adjusted for M2+ Lg BNP and LVEF; Model 4: Adjusted for M3+ TyG-NLR.

The prognostic value of the TyG-NLR for all-cause mortality in patients with heart failure was studied by generating ROC curves (Figure 2). The ROC curve revealed areas under the curve (AUCs) of 0.570 (95% CI: 0.536–0.605, *P* < 0.05) for the TyG, 0.720 (95% CI: 0.690–0.752, *P* < 0.05) for the NLR, and 0.723 (95% CI: 0.693–0.753, *P* < 0.05) for the TyG-NLR, with the AUC for the TyG-NLR being the largest (*P* < 0.05). And the optimal cut-off value of TyG-NLR is 7.48, with a sensitivity of 65.3% and a specificity of 79.4%. Thus, the combination of the TyG index and NLR was superior to the TyG index or NLR alone in the prediction of HF.

ROC curve analysis revealed that the AUC of Model 1 for predicting all-cause mortality in HF patients was 0.640 (95% CI: 0.612–0.661, *P* < 0.05). Model 2 predicted all-cause mortality in HF patients with an AUC of 0.727 (95% CI: 0.698–0.746, *P* < 0.05). Model 3 had an AUC of 0.759 (95% CI: 0.723–0.883, *P* < 0.05). Model 4 predicted all-cause mortality in HF patients with an AUC of 0.802 (95% CI: 0.785–0.846, *P* < 0.05), as shown in Figure 2. The results show that the model with the TyG-NLR is superior to the traditional model in predicting all-cause mortality in patients with HF and has particular significance in guiding clinical work.

**Figure 3:**
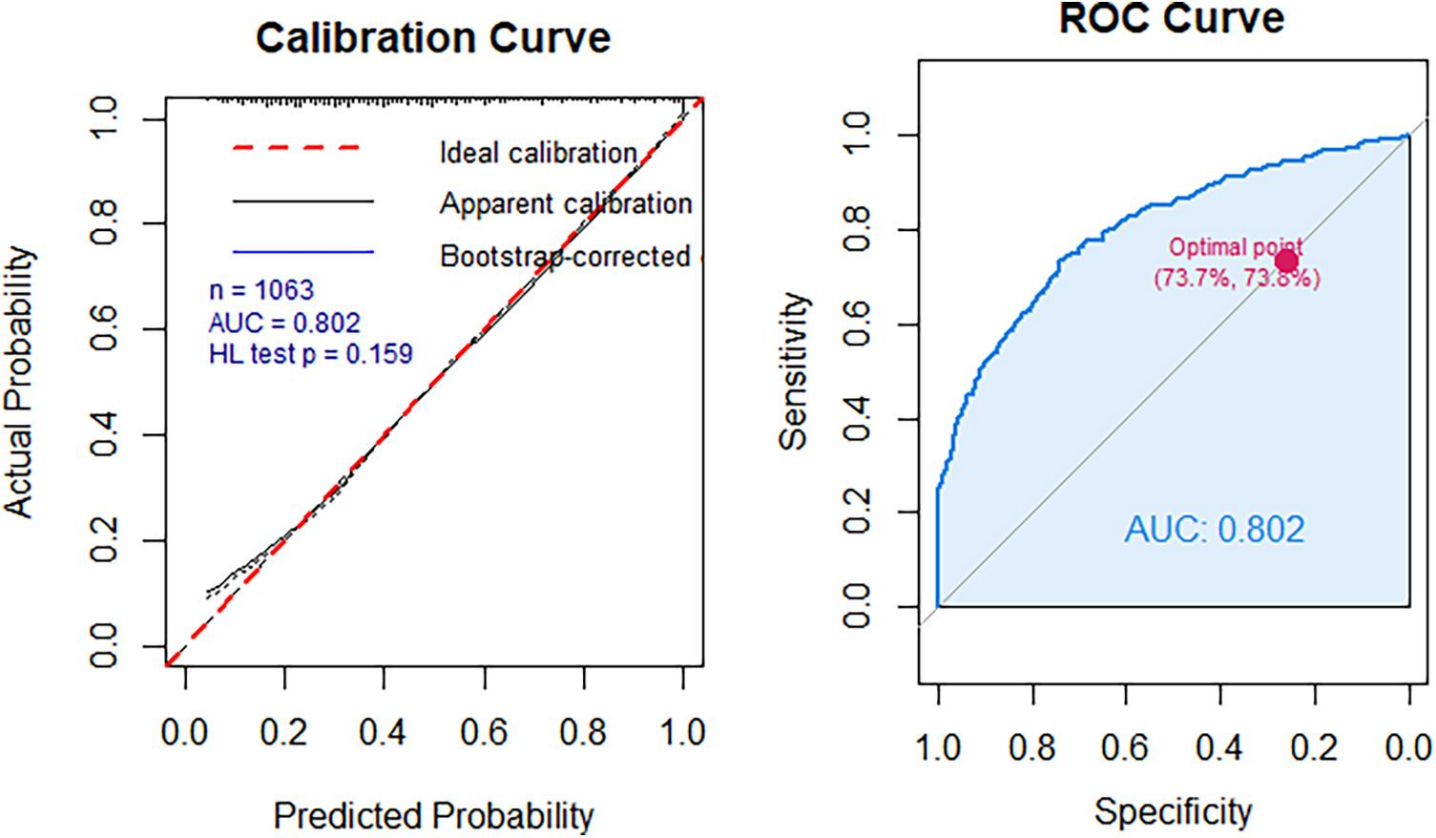
The calibration of a clinical prediction model with and without TyG-NLR.

**Figure 4:**
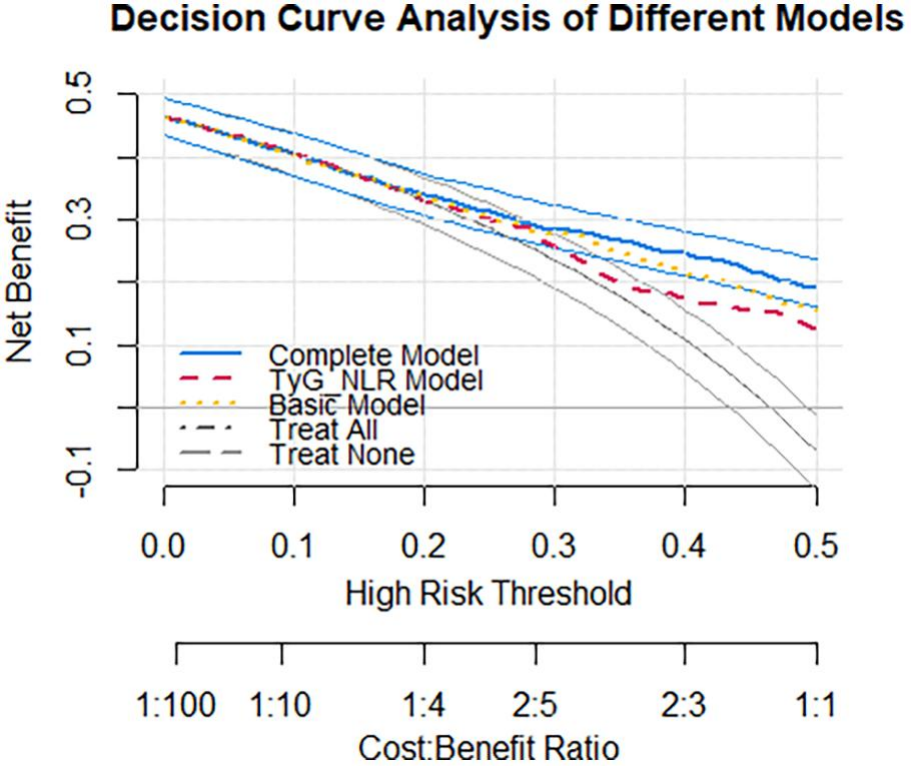
Decision curve analysis: assessment of net benefit of TyG-NLR in predicting all-cause mortality. Basic model adjustment included: age, sex, BMI, serum chloride, sodium, albumin, fibrinogen, Lg BNP and LVEF. Complete model adjustment included: Basic model + TyG-NLR. TyG-NLR model: unadjusted. Treat None: No intervention strategy; All, Assume all individuals are treated.

We assessed the calibration of a clinical prediction model with and without TyG-NLR using calibration plots and the Hosmer-Lemeshow (HL) test (Figure 3). The model incorporating TyG-NLR showed good calibration (*p* = 0.159 for the HL test). We performed net reclassification improvement (NRI) and integrated discrimination improvement (IDI) analyses (Table 3). Adding TyG-NLR to the basic model (i.e., Model 3) yielded a significant continuous NRI of 0.468 (95% CI: 0.355–0.586, *p* < 0.001) and an IDI of 0.085 (95% CI: 0.068–0.105, *p* < 0.001), indicating improved risk stratification. We performed decision curve analysis (DCA) to evaluate the clinical net benefit. As shown in Figure 4, across a range of clinically reasonable risk thresholds, the model containing TyG-NLR provided a higher net benefit than both the “treat-all” and “treat-none” strategies, as well as the basic model without TyG-NLR.

**Table 3:** Incremental predictive value of TyG-NLR.

Model	HR (95% CI)*	AUC (95% CI)	IDI (95% CI)	cNRI (95% CI)
Basic model + TyG-NLR	1.584 (1.285, 1.952)	0.802 (0.785, 0.846)	0.085 (0.068, 0.105)	0.468 (0.355, 0.586)

Basic model adjustment included: age, sex, BMI, serum chloride, sodium, albumin, fibrinogen, Lg BNP and LVEF.

* The basic model adjustments in the Cox proportional hazards models additionally include: TyG and NLR.

### 3.5 Subgroup analysis

According to the unadjusted Cox analysis, the TyG index and NLR were independent predictors of all-cause death in patients with HF. (TyG: HR: 2.531, 95% CI: 2.101–3.049, *p* < 0.001; NLR: HR: 2.746, 95% CI: 2.250–3.315, *p* < 0.001). After some confounding factors were corrected, the TyG index and NLR remained independent predictors, as shown in Table 4.

**Table 4:** Subgroup analysis.

	Unadjusted		Adjusted	
	HR (95% CI)	*p*	HR (95% CI)	*p*
TyG < 1.79	Ref.		Ref.	
TyG ≥ 1.79	2.531 (2.101, 3.049)	<0.001	1.324 (1.088, 1.611)	0.005
NLR < 3.31	Ref.		Ref.	
NLR ≥ 3.31	2.746 (2.25, 3.315)	<0.001	1.903 (1.557, 2.326)	<0.001
Combined categories				
Group 1: TyG < 1.79 + NLR < 3.31	Ref.		Ref.	
Group 2: TyG < 1.79 + NLR ≥ 3.31	2.723 (2.023, 3.666)	<0.001	2.033 (1.499, 2.758)	<0.001
Group 3: TyG ≥ 1.79 + NLR < 3.31	1.432 (1.041, 1.970)	0.027	1.486 (1.072, 2.060)	0.018
Group 4: TyG ≥ 1.79 + NLR ≥ 3.31	4.377 (3.262, 5.875)	<0.001	2.984 (2.164, 4.114)	<0.001

Adjusted for age, sex, BMI, coronary heart disease, hypertension, diabetes, serum chloride, sodium, albumin, fibrinogen, Lg BNP and LVEF.

Pearson's correlation analysis revealed that the TyG index was positively correlated with the NLR (*r* = 0.918, *P* < 0.0001). Cox regression revealed an interaction effect between the TyG index and the NLR, so subgroup analysis was performed to determine the association. All patients with HF were divided into 4 groups (Group 1: TyG < 1.79 + NLR < 3.31; Group 2: TyG < 1.79 + NLR ≥ 3.31; Group 3: TyG ≥ 1.79 + NLR < 3.31; and Group 4: TyG ≥ 1.79 + NLR ≥ 3.31). Group 1 was set as the reference group, and HR values were calculated. Subgroup analyses revealed that after adjusting for age, sex, BMI, coronary heart disease, hypertension status, diabetes status, serum chloride, sodium, albumin and fibrinogen levels, Lg BNP, and LVEF, all-cause mortality rates were 2.033 times higher in Group 2 (*p* < 0.001), 1.486 times higher in Group 3 (*p* = 0.018), and 2.984 times higher in Group 4 (*p* < 0.001) (Table 4).

## 4 Discussion

This is the first study to investigate the relationship between the TyG-NLR and long-term prognosis in patients with HF. Our findings demonstrate that a higher TyG-NLR index, integrating insulin resistance and systemic inflammation, was independently associated with increased all-cause mortality in patients with HF and showed incremental predictive value over established risk factors. The cumulative probability of all-cause mortality was significantly greater in those with high TyG index values and NLRs. Therefore, the TyG-NLR could serve as a simple and cost-effective marker for risk stratification and early detection of individuals at increased risk for HF. Compared with those in the low TyG-NLR group, the patients in the high TyG-NLR group had higher neutrophil counts, higher TG levels, lower lymphocyte counts, and lower GFR levels. By univariate and multivariate Cox proportional hazards analyses, we identified the TyG-NLR as an independent predictor of all-cause mortality in patients with CHF. In the subgroup analysis, the risk of death and all-cause mortality rates were 2.033 times higher in Group 2 (*p* < 0.001), 1.486 times higher in Group 3 (*p* = 0.018), and 2.984 times higher in Group 4 (*p* < 0.001) when Group 1 was set as the reference group. In addition, the ROC curve analysis revealed that the model with the TyG-NLR is superior to the traditional model in predicting all-cause mortality in patients with HF and. Calibration, reclassification, and decision curve analyses show that the TyG-NLR changes risk estimates in a way that matters at the bedside. While the NRI and IDI show significant statistical improvement, and the DCA suggests potential net benefit across a range of thresholds, this does not equate to immediate clinical implementation. These findings highlight the potential pathophysiological and prognostic relevance of the metabolic-inflammatory interplay in HF, but the TyG-NLR index is not yet ready for routine clinical application. Its clinical utility requires rigorous external validation in diverse populations and healthcare settings, as well as prospective evaluation to determine if it meaningfully improves upon existing heart failure risk prediction models and guides therapy.

IR is a well-recognized factor with multiple noxious effects, including endothelial dysfunction, oxidative stress, and myocardial remodeling, all of which contribute to impaired cardiac function and the development and progression of HF (6, 18). Several methods to assess IR have been developed, and among these methods, the HOMA-IR and the TyG index are considered valuable and reliable markers of IR (7, 8). Homeostasis model assessment (HOMA) has been used as a relatively simple and reliable method for assessing IR in previous studies (27, 28). With the continuous deepening of TyG index studies, the TyG index has been confirmed to have a strong association with the gold standard test for the diagnosis of IR. Its predictive power is greater than that of the IR homeostasis assessment model (29, 30). Kelly et al. demonstrated that serum TG levels were negatively correlated with insulin sensitivity, suggesting that the homeostasis between lipid metabolism and insulin efficacy is disrupted with increasing TG levels (31). Sanchez-Garcia A et al.24 reported that the TyG index had a sensitivity of 96% in the diagnosis of IR in a meta-analysis of 69,922 patients in 15 studies (32). In a study conducted on Chinese diabetic patients with a high BMI, the TyG index (cutoff of 7.99) was able to identify IR more effectively than the HOMA-IR index (cutoff of 3.39) (33).

The TyG index is positively associated with diabetes and increased risk of metabolic and atherosclerotic cardiovascular diseases (34, 35). Li and coworkers, through an analysis of two large Chinese cohorts (a total of 115,341 subjects), demonstrated that a high TyG index was an independent and causal risk factor for incident HF (18). Our research results are consistent with these findings. A higher TyG index is directly related to impaired left ventricular (LV) structure and function, augmented myocardial fibrosis, and an increased risk of HF (16, 20, 36). Xu et al., based on a 9-year follow-up cohort, revealed a positive association between the TyG index and initial-onset HF, which could be attributed to insulin resistance (IR) (20). Furthermore, to study whether and how the TyG index can be a useful prognostic indicator for HF patients, Cheng et al. examined 886 nondiabetic patients with AHF and reported significantly higher in-hospital mortality in those patients with higher TyG index values (37). The link between insulin resistance, as assessed by the TyG index, and the development of HF may involve multiple mechanisms. First, a higher TyG index reflects a state of insulin resistance associated with metabolic imbalances, including hyperglycemia and dyslipidemia, and has a direct effect on cardiomyocyte hypertrophy, myocardial contractility, and cardiac stiffness (38–40). Second, insulin resistance promotes the progression of chronic inflammation (41) and endothelial dysfunction (42), facilitates the formation of vulnerable plaques, and plays an important role in the pathogenesis of cardiomyocyte apoptosis and myocardial fibrosis, weakening myocardial compensatory mechanisms and increasing susceptibility to ischemia and pressure overload (43). Third, insulin resistance is associated with increased activity of the sympathetic nervous system and the renin–angiotensin–aldosterone system, extracellular matrix deposition, and intramyocardial lipid deposition, leading to myocardial fibrosis and cardiac dysfunction (44–46). This study also revealed that higher TyG index values were associated with higher HRs of HF incidence.

The NLR is a prognostic marker for hospitalization and death in HF patients. The NLR, which is calculated from neutrophil counts and lymphocyte counts, reflects the balance between the body's intrinsic (i.e., neutrophil) and adaptive (i.e., lymphocyte) immune responses. There are several possible reasons for why a higher NLR increases all-cause mortality in patients with heart failure. Neutrophils release a variety of proteolytic enzymes, such as elastase, acid phosphatase, and myeloperoxidase, with devastating effects on cardiac tissue (47, 48). The secretion of these inflammatory signals, coupled with the increased release of granulocyte–monocyte colony–stimulating factor, hypoxia signals, etc., during inflammation ultimately prolongs the lifespan of neutrophils and adversely affects the heart (49, 50). A study performed by Arruda-Olson demonstrated that myocardial infarction patients with neutrophilia were independently connected with increased occurrence of heart failure and all-cause mortality (51). On the other hand, possible mechanisms of lymphopenia include lymphocyte apoptosis, downregulation of lymphocyte differentiation and proliferation, and neurohumoral activation (52, 53). Related studies have shown that inflammation leads to increased lymphocyte apoptosis. HF, as a state of stress and activation of the hypothalamic–pituitary–adrenal axis, leads to increased secretion of adrenal cortisol. This hormone induces lymphocyte apoptosis and subsequent lymphopenia (54–56). In addition, TNF-α is thought to be responsible for the decrease in lymphocyte counts (57). The NLR is considered to be a better tool for predicting the death of HF patients than its independent components (neutrophils and lymphocytes).

The study by Bećirović et al. (58) provides valuable evidence on the differential levels of systemic inflammation across heart failure phenotypes, demonstrating that NLR is significantly elevated in HF. A retrospective study by Angkananard et al. revealed that an elevated NLR on admission was independently associated with more severe cardiovascular events, HF rehospitalization, and in-hospital death in 321 patients with acute HF (59). The NLR also has broad applicability and risk representation in other disease states, including several cancers (60, 61), irritable bowel syndrome (62), and spontaneous cerebral hemorrhage (63). Although the exact mechanism of the influence of NLR increase on prognosis remains to be clarified, it may be related to an increase in neutrophil-dependent inflammation and a decrease in the lymphocyte-mediated inflammatory response.

In summary, the TyG index and NLR are independent prognostic factors for HF. Lin et al. compared the effects of TyG index and NLR on the prognosis of metabolic syndrome in healthy people in China (64). Zhang et al. reported that, compared with the TyG index or NLR, the combination of the TyG index and NLR is beneficial for improving the diagnostic accuracy of CAD and CAD severity (65). Wang's study showed that the TyG score combined with the NLR could reasonably predict the occurrence of MACE after PCI in STEMI patients and the clinical utility of the prediction model (66). In a study by Guo et al. (67) involving adult patients with type 2 diabetes, TyG-NLR was independently associated with cardiorenal disease severity. TyG-NLR demonstrated a steeper risk gradient and modest improvements in discrimination and calibration, and it yielded slightly higher net clinical benefit across clinically relevant decision thresholds. Therefore, this study explored the value of the TyG index combined with the NLR in the prognosis of heart failure patients. Our research revealed that an increased TyG-NLR is an independent predictor of increased mortality in patients with HF.

The performance of the TyG-NLR index should be evaluated in relation to established heart failure risk prediction models. In comparison with widely validated models such as the MAGGIC risk score and the Seattle Heart Failure Model, the TyG-NLR index offers distinct advantages in accessibility, ease of calculation, and low cost, as it relies solely on routine laboratory parameters. These characteristics may support its potential utility in rapid screening or resource-limited settings. However, several important limitations of the present study should be acknowledged. Most notably, there is currently no evidence that the TyG-NLR index provides superior predictive performance for hard endpoints—such as all-cause mortality and heart failure rehospitalization—compared with existing complex models, nor that it can replace them. Therefore, at this stage, the TyG-NLR index should be considered a promising research tool or a useful complement to existing risk-stratification systems, rather than a direct substitute. Future prospective, multicentre studies are warranted to validate its performance in broader populations and to further evaluate its practical value for improving clinical decision-making and patient outcomes.

## 5 Conclusion

Our study reveals the potential value of TyG-NLR as a cost-effective and sensitive biomarker in understanding and predicting heart failure. An increased TyG-NLR is an independent predictor of increased mortality in patients with HF.

## 6 Limitations

This study has several limitations. First, its retrospective, single-center design may introduce selection bias and unmeasured confounding factors. Although we adjusted for a wide range of clinical variables, residual confounding cannot be entirely ruled out. Second, the association between the TyG-NLR index and adverse prognosis was observed in a specific cohort of hospitalized patients with acute decompensated heart failure; therefore, the findings may not be generalizable to stable outpatients or other clinical settings. Third, the use of a data-driven median cutoff for TyG-NLR carries a risk of overfitting, and its generalizability remains uncertain. Thus, the findings should be considered hypothesis-generating, and the identified cutoff should be viewed as a candidate threshold requiring external validation. Further prospective, multicenter studies involving independent cohorts are needed to confirm the prognostic value and clinical applicability of the TyG-NLR index. Finally, as the study primarily included patients with NYHA class III or IV heart failure, the results may not be applicable to populations with mild to moderate symptoms.

## Data Availability

The raw data supporting the conclusions of this article will be made available by the authors, without undue reservation.
