# Supplementary figures and images for: Prognostic significance of the TyG index combined with the NLR for heart failure: a retrospective study
Source: Front Cardiovasc Med. 2026 Apr 21;13:1712467. doi: 10.3389/fcvm.2026.1712467 (PMC13139348; doi:10.3389/fcvm.2026.1712467)

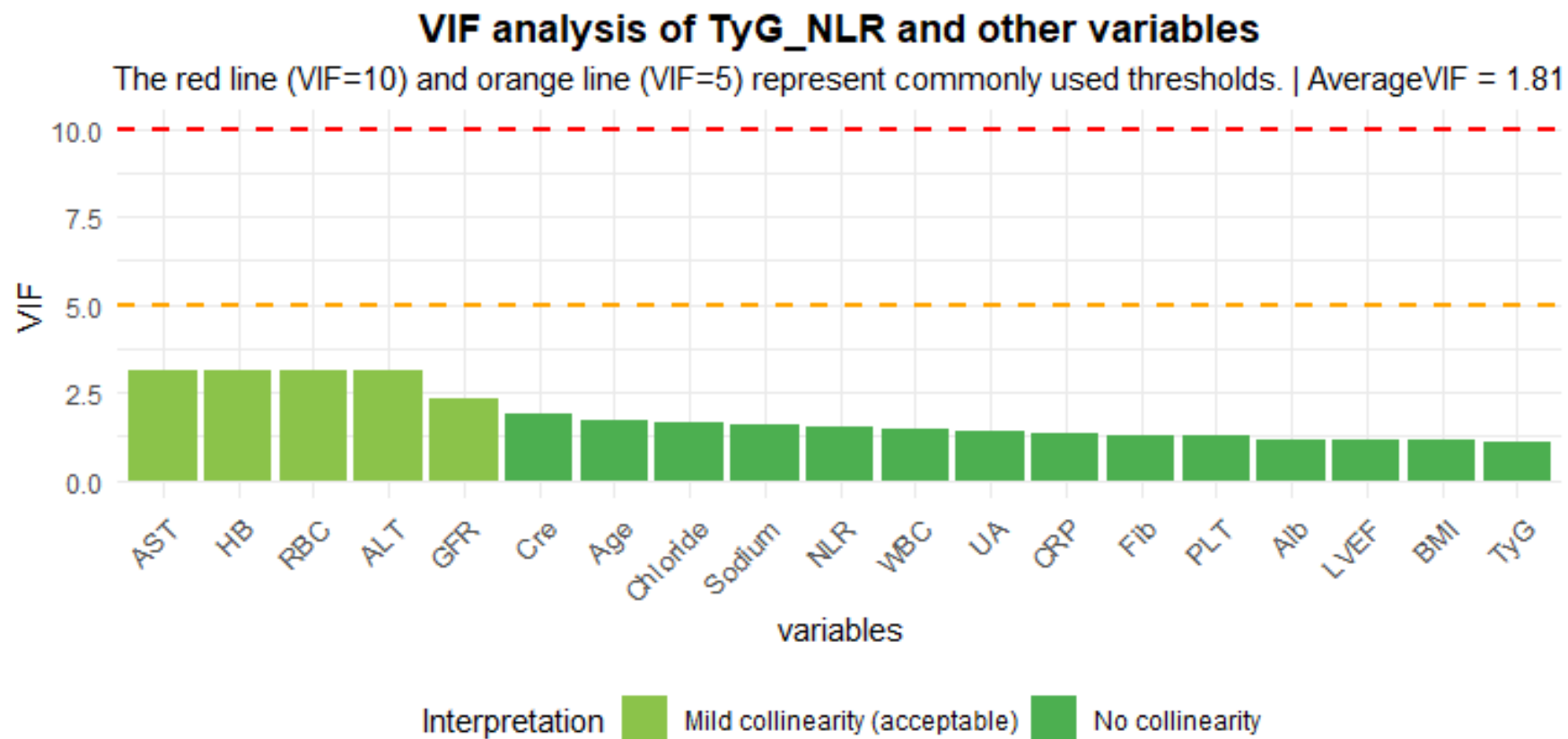

Supplementary Figure 1. VIF analysis of TyG-NLR and other variables.

Supplement: Supplementary file 1 [file Image1.pdf]
